# Reward disrupts reactivated human skill memory

**DOI:** 10.1038/srep28270

**Published:** 2016-06-16

**Authors:** Eran Dayan, Rony Laor-Maayany, Nitzan Censor

**Affiliations:** 1 National Institute of Neurological Disorders and Stroke, National Institutes of Health, Bethesda, MD 20892, USA; 2 School of Psychological Sciences and Sagol School of Neuroscience, Tel-Aviv University, Tel Aviv 69978, Israel

## Abstract

Accumulating evidence across species and memory domains shows that when an existing memory is reactivated, it becomes susceptible to modifications. However, the potential role of reward signals in these mechanisms underlying human memory dynamics is unknown. Leaning on a wealth of findings on the role of reward in reinforcing memory, we tested the impact of reinforcing a skill memory trace with monetary reward following memory reactivation, on strengthening of the memory trace. Reinforcing reactivated memories did not strengthen the memory, but rather led to disruption of the memory trace, breaking down the link between memory reactivation and subsequent memory strength. Statistical modeling further revealed a strong mediating role for memory reactivation in linking between memory encoding and subsequent memory strength only when the memory was replayed without reinforcement. We suggest that, rather than reinforcing the existing memory trace, reward creates a competing memory trace, impairing expression of the original reward-free memory. This mechanism sheds light on the processes underlying skill acquisition, having wide translational implications.

It is by now widely accepted that our memories are dynamic, even after their initial stabilization through consolidation. Thus, once an existing, already consolidated memory is retrieved or reactivated by a reminder, it becomes susceptible to modification^1^,^2^,^3^,^4^,^5^. The reactivation and reinstatement of a memory trace can degrade^1^,^3^,^6^,^7^, or conversely stabilize or strengthen^4^,^8^ it through the proposed mechanisms of reconsolidation^9^,^10^. However, despite an ever increasing interest in memory reactivation and reconsolidation, originating from the far-ranging translational implications of this line of research, our understanding of the mechanism that leads to memory modifications remains limited. As recent research has identified the unique role of reward and of reinforcement mechanisms in episodic and procedural memory formation^11^,^12^,^13^,^14^,^15^,^16^,^17^, here we set out to uncover how reinforcement contributes to memory modification through reactivation. In the first experiment we had a group of subjects learn a sequential motor skill^18^,^19^,^20^ over 12 blocks of training. Then, a day later, subjects performed one reactivation trial of the task, with half of them receiving monetary reward in accordance with their performance and the rest receiving performance feedback alone. Memory strength was then measured on day 3 for all subjects, focusing on the specific mediating role of reactivation in the link between memory encoding and subsequent memory strength. An additional experiment tested the time-dependent effects of reinforcement following reactivation.

## Results

**Figure 1:**
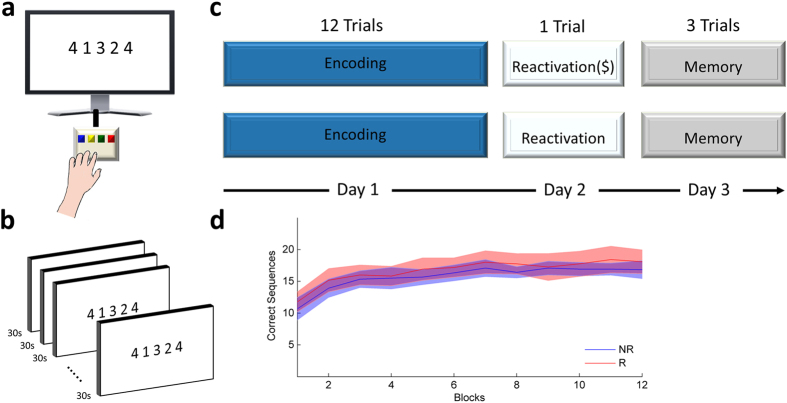
Task and design. (**a**) Subjects performed a sequential finger-tapping task where they had to repeatedly tap with their left non-dominant hand a 5-element sequence of finger movements as quickly and accurately as possible. Performance in the task was quantified in terms of the number of correct sequences tapped during each trial. (**b**) The task was composed of separate trials, lasting 30 s, separated by 30 s breaks. (**c**) The experiment included three sessions. In the first session (Day 1) all subjects performed twelve trials of the sequential finger-tapping task. On the next session (Day 2), participants were divided into two groups, the first performing one reactivation trial with reinforcement and the second with performance feedback instead of reinforcement, allowing to tease apart monetary reward and simple performance feedback per se. In the third session (Day 3) subsequent memory strength was measured by having both groups perform three test trials of the task, with no performance or reward feedback. (**d**) Both the rewarded and unrewarded groups showed comparable encoding of the skill memory. Shaded lines denote standard errors of the mean.

We had subjects (n = 24) perform a sequential finger-tapping motor memory task^18^,^19^,^20^, where they were asked to tap a sequence of key presses, as quickly and as accurately as possible during trials lasting 30 s each (Fig. 1a). An Initial 12 trials encoding phase took place on the first day of the experiment (Fig. 1b). Subjects then returned a day later, and were assigned to one of two groups. The first group (the *rewarded group*) was administered one reactivation trial of the task, with the number of correct sequences tapped during the trial monetarily reinforced. The second group of subjects (the *unrewarded group*) was administered the same reactivation trial, receiving feedback about their performance, rather than monetary reward (Fig. 1c). This design allowed us to tease apart monetary reward and simple performance feedback per se. All subjects then returned a day later and performed 3 additional trials, where their memory strength for the acquired skill was measured (Fig. 1c). Repeated measures analysis of variance (ANOVA) indicated that both groups showed comparable encoding (ΔDay1_end_ -Day1_start_) of the skill memory (F_1,22_ = 0.536, ns, Fig. 1d). Of note, the groups also showed comparable reactivation strength (ΔDay2-Day1_end_, t_22_ = −1.017, ns).

**Figure 2:**
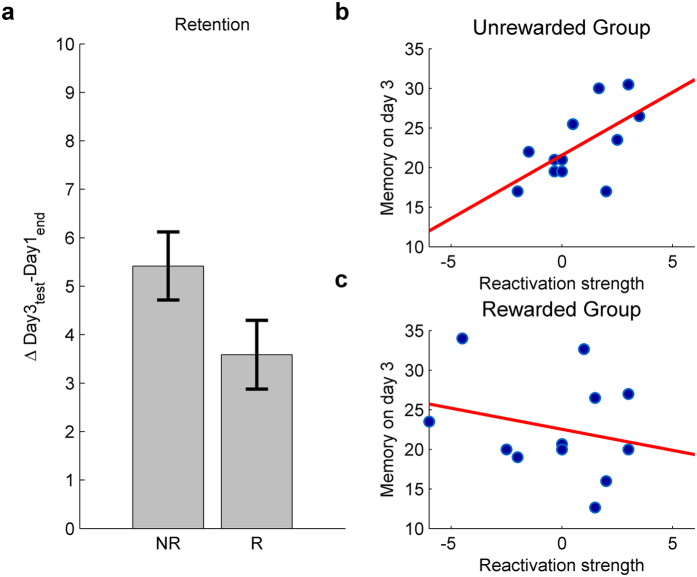
Effects of reactivation on subsequent memory strength. (**a**) Retention for the skill memory was inferior in the rewarded group. R, rewarded group; NR, unrewarded group. (**b**) In the unrewarded group reactivation strength significantly correlated with memory on Day 3 test (r = 0.616, p = 0.017). (**c**) In the rewarded group the correlation between memory reactivation strength and memory strength on Day 3 was not significant (r = −0.243, ns) and was significantly weaker than in the unrewarded group. Error bars denote standard errors of the mean.

Not only that applying reward following reactivation did not strengthen (ΔDay3_test_-Day1_end_) memory retention compared to unrewarded reactivation (t_22_ = −1.834, ns), (Fig. 2a), but rather it exhibited a tendency towards inferior retention. To better understand the role of reward in skill memory reactivation we next examined whether the strength of reactivation correlated with subjects’ subsequent memory strength on Day3. Whereas a statistically significant correlation was found between reactivation strength and memory strength on Day3 in the unrewarded group (r = 0.616, p = 0.033, Fig. 2b), this correlation was abolished in the rewarded group (r = −0.243, ns, Fig. 2c). Both correlations were robust against outliers (bend correlation r = 0.606 and r = −0.08 in the unrewarded and rewarded groups respectively), and significantly different from one another (Fisher Z = 2.05, p = 0.0404).

**Figure 3:**
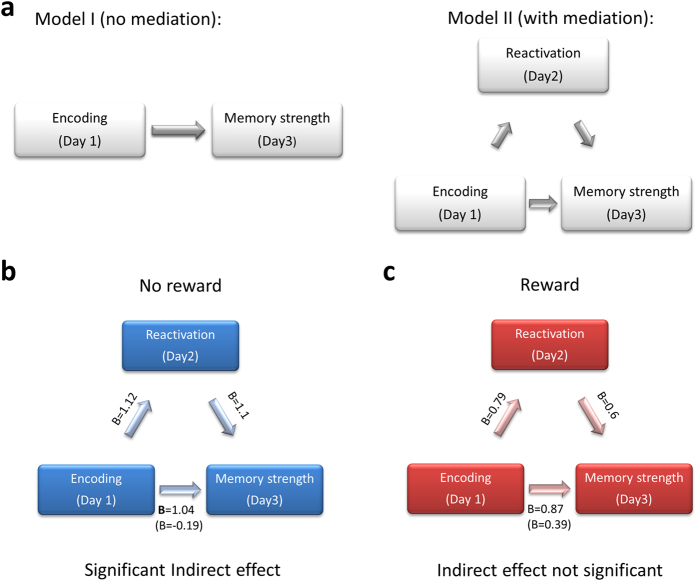
The mediating role of reactivation in the link between encoding and subsequent memory strength. (**a**) We considered two different models for the links between encoding and Day 3 memory strength, a first (Model I) assuming a direct link between the two, and a second indirect model (Model II) where reactivation mediates this link. (**b**) In the unrewarded group the link between encoding and Day 3 memory strength was mediated by reactivation. (**c**) In the rewarded group, reactivation failed to mediate the link between encoding and day 3 memory strength. B weights (regression coefficients) are shown for each path in the model (in brackets is the path from encoding to Day 3 memory strength when the mediator is also included in the model).

To further uncover the effect of reward on memory reactivation and subsequent memory strength we considered two competing models. According to the first, the magnitude of memory on Day 3 depends on how well the memory was encoded, regardless of reactivation (Fig. 3a). In a second, indirect model, the effects of encoding on Day 3 memory strength are mediated by the strength of reactivation (Fig. 3a). Modeling the behavior displayed by the rewarded and unrewarded groups revealed clear differences among the two. Regression analysis indicated an overall similar strength, among the two groups, in each of the direct paths in the model (i.e., encoding to Day 3 memory strength, encoding to reactivation and reactivation to Day 3 memory strength; Fig. 3b,c, Table 1). However, testing for mediation effects based on an indirect path from encoding to Day 3 memory strength, bias-corrected confidence estimates and bootstrap resampling^21^,^22^ showed that in the unrewarded group the effect of encoding on Day 3 memory strength was significantly mediated by reactivation (B = 1.284, confidence interval = 0.241 to 2.849, Table 2), but not in the rewarded group (B = 0.469, confidence interval = −0.23 to 1.01, Table 2).

**Figure 4:**
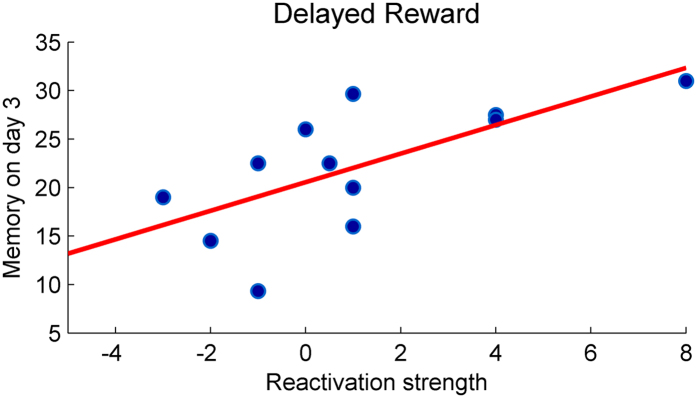
Relation between the strength of memory reactivation and Day 3 memory strength in delayed reward conditions. Reactivation strength significantly correlated with Day 3 memory strength (r = 0.678, p = 0.015).

To separate between the effects of reactivation and the effects of reward per se and to control for possible intervening effects stemming from the timing of reinforcement, we ran a second experiment in which subjects (n = 12) underwent the same experimental procedures, wherein performance on the reactivation trial was reinforced with a fixed delay of 6 h, a period which may surpass the time-window during which memory becomes susceptible to further modifications following reactivation^10^. In this *delayed reward* condition reactivation strength significantly correlated with Day 3 memory strength (r = 0.678, p = 0.015; Fig. 4), in a way which did not differ from the unrewarded group (Z = 0.23, ns). Additionally, retention did not differ from that of the unrewarded group (t_22_ = 1.527, ns). Overall, these results suggest that reinforced memory reactivation disrupts subsequent memory strength.

## Discussion

The goal of this study was to identify the role of reinforcement in reactivated skill memory. As reward has been shown to mediate the encoding of information in both the procedural and declarative memory systems^11^,^12^,^13^,^14^,^15^,^16^,^17^, we reasoned that reinforcing an already encoded procedural memory following reactivation would impact strengthening of the memory trace and facilitate retention. We report however that reactivation with reward did not strengthen memory, relative to reactivation with no reward. Moreover, the relation between the strength of memory reactivation and subsequent memory as found in reward-free reactivation, was reduced for reward-based reactivation. In addition, statistical modeling revealed that unrewarded, as opposed to rewarded reactivation, indirectly mediated the link between encoding and subsequent memory strength.

A viable framework for interpreting the current results is that of competition between memory traces and memory systems. It is by now well accepted that various tasks invoke competition between memory systems that rely on dissociable brain networks and distinct computational processes^17^,^23^,^24^,^25^. Competitive memory dynamics can also be formed between memory traces, and may specifically originate from reinforcement mechanisms. For instance, encoding of reward associated items interferes with the encoding of non-reinforced mnemonic representations^25^. Moreover, in episodic memory, competitive dynamics between memory encoding and learning from reward has been documented^17^, consistent with differential engagement of the medial temporal lobes and the striatum during learning^25^. Thus, a feasible framework for interpreting the disruptive effect of reinforcement following reactivation on subsequent memory strength is that the introduction of reward following reactivation may have resulted in a competition with the original encoded trace, which was averted when the memory trace was replayed with no reward. This framework is consistent with findings in Pavlovian learning demonstrating retroactive interference of new memories on reinstated memories^26^,^27^,^28^, or modification of reactivated memories through counterconditioning with new reinforcers^29^,^30^,^31^.

An alternative but related explanation on the unfavorable effects of reinforcement following reactivation on subsequent memory strength is based upon a prediction error mechanism. Prediction errors are believed to be a prerequisite for memory destabilization and reconsolidation^32^,^33^,^34^. However, it was recently suggested that memories can be weakened when they are mispredicted by the context, which was originally associated with these memories^35^. The current results may reflect a related prediction error mechanism, whereby the addition of reinforcement during the replay of procedural memory generates a prediction error which subsequently weakens memory. This prediction error is absent when the memory trace is replayed with no reward, leading to superior subsequent memory. Thus, in this respect reward may generate a previously unencountered context which ultimately weakens the memory trace. Future research should take into account that a combination of memory competition and prediction error mechanisms may underlie the disruptive effect reward has on the reactivated skill memory, both generating an impairment in subsequent memory strength. Our results further indicate that delaying the receipt of reward after the reactivation trial results in an intact significant relation between reactivation strength and subsequent memory strength, comparable to unrewarded reactivation. Thus, our results point to time dependent effects of reward following reactivation, on subsequent memory strength.

Taken together, these results open interesting avenues for future research. First, it remains to be tested if extrinsic modulation of reward systems, whether by means of pharmacological interventions^36^,^37^, or using non-invasive brain stimulation^38^ exerts a similar influence on memory reactivation and subsequent memory strength. This will allow to further uncover the role of dopaminergic neuromodulation during memory reactivation. Second, the putative competitive dynamics between memory traces and their underlying neural underpinning could be further tested using brain imaging techniques, suitable for probing complex information representations, such as multivariate pattern classification analysis^39^,^40^.

The notion that existing memories can be modified with external interventions has far reaching clinical implications, as such interventions can be employed, for instance to disrupt maladaptive memories after post-traumatic stress or to reduce drug craving in addiction^41^. The current results demonstrate the contextual specificity required for these interventions to as found and point to the need for additional studies, to further delineate the role of memory reactivation in shaping subsequent memory strength.

## Materials and Methods

### Subjects

A total of thirty-six right-handed healthy subjects (13 men, 23 women; mean age 24.8 ± 2.2 standard deviation) participated in the study. All subjects gave their written informed consent, approved by Tel Aviv University’s Ethics committee. All procedures were in accordance with approved guidelines. Musicians (in the past or present) were excluded from participating in the study. We have additionally required at least 6 h of sleep prior to each experimental session.

### Task

During the experiment subjects were asked to perform a sequential finger-tapping task^18^,^19^,^20^,^42^. Each trial in the task lasted 30 sec, during which subjects had to repeatedly tap with their left non-dominant hand a 5-element sequence of finger movements as quickly and accurately as possible (the sequence was 4-1-3-2-4, whereby ‘1’, ‘2’, ‘3’ and ‘4’ correspond to tapping of the index, middle, ring and little fingers respectively). Tapping was performed on a 4-key response box (Cedrus, Lumina, Model LU440), placed in front of subjects during the experiment. Performance in the task was quantified in terms of the number of correct sequences tapped during each trial^18^,^19^,^42^. The same sequence was used in all experiments and sessions. Throughout each trial, each key press produced a dot displayed at the top portion of the screen, with the dots accumulating from left to right as the trial progressed. Trials were separated by 30 s breaks.

### Experimental procedure

The first experiment comprised three sessions, administered on three consecutive days. In the first session (Day 1) all subjects (n = 24) performed twelve trials of the sequential finger-tapping task. On the next session (Day 2), which was administered 24 hours later, participants in the main experiment were equally divided into two groups. The first, rewarded group, performed one reactivation trial whereby each successful sequence within the trial was reinforced with monetary reward. Subjects were explicitly told that they will be reinforced at the end of the trial. The total reward earned in the trial was displayed on the screen right after the completion of the trial (indicating: “you won X Shekels!” with X being the amount of Israeli Shekels that is equal to the number of total correct sequences performed in the trial). Instructions given to this group of subjects prior to the task explicitly indicated that they will be monetarily rewarded with 1 Israeli Shekel for each of the correct sequences they perform during the task. A second, unrewarded group, performed one reactivation trial with no monetary reward, however this group of subjects received performance feedback at the end of the trial indicating how many successful sequences they were able to tap during the trial (“you tapped X correct sequences!”). Subjects were explicitly told that they will receive performance feedback at the end of the trial. This design enabled to tease apart monetary reward and simple performance feedback per se. Thus, both groups were administered a reactivation trial at Day 2, eliminating differences resulting from retrieval-induced forgetting^43^,^44^. In the third session (Day 3) all participants performed three regular trials of the task, with no performance or reward feedback. In a second experiment, subjects (n = 12) were reinforced in accordance with their performance in the reactivation trial, similar to the rewarded group. However in this experiment reward feedback was provided 6 hours after the completion of the reactivation trial (in the same way as the reward group). Instructions in this experiment indicated to the subjects that they will “receive feedback” about their performance 6 hours after the reactivation trial (i.e., the subjects were not aware that their performance will be reinforced with monetary reward).

### Data analysis

To test for group differences in encoding, a repeated measures analysis of variance (ANOVA) was performed with group serving as the between-subjects factor, and the first and last three trials of Day 1 as repeated measures. The ANOVA was preceded by Mauchly test of sphericity, to confirm that the assumption of sphericity was not violated. Retention was defined as the difference in performance between Day 3 and the last 3 trials of Day 1. As in previous studies using the same task, to better characterize memory strength by minimizing motor fatigue-related decrements in performance, the best two trials were considered for Day 1 post-training and for Day 3 memory strength^45^,^46^. Two tailed tests were used in all analyses. The relationship between reactivation strength (defined as the difference in performance between Day 2 reactivation and Day 1 post-training), and Day 3 memory strength was tested with a Pearson’s correlation. We have additionally assessed the robustness of these correlations against outliers using the percentage-bend correlation technique^47^, as implemented in the “Robust Correlation Toolbox” in MATLAB^48^. Group differences in the strength of correlation were tested with a Fisher’s r-to-z transformation.

Mediation effects were tested using regression analysis and bias-corrected confidence estimates. First, simple linear regression analyses between each component in the mediation model were performed, including the effect of Day 1 encoding on Day 2 reactivation (A path), Day 2 reactivation on Day 3 subsequent memory strength (B path), Day 1 encoding on Day 3 memory strength (C path), and encoding on Day 3 memory strength when the putative mediator (Day 2 reactivation) is also in the model (the C’ path). In this model, the indirect effect estimates the degree to which encoding exerts an indirect effect on Day 3 memory strength through reactivation (the mediator). Mediation effects (the indirect path) were tested using bootstrapping with bias-corrected confidence estimates^21^,^49^, defining confidence interval (99% to account for multiple comparisons) with 5000 bootstrap resamples^22^. Confidence intervals that included the value 0 indicated that the null hypothesis (no mediation effects) could not be rejected whereas intervals that did not include 0 indicated that the null hypothesis should be rejected.

## Additional Information

**How to cite this article**: Dayan, E. *et al*. Reward disrupts reactivated human skill memory. *Sci. Rep*. **6**, 28270; doi: 10.1038/srep28270 (2016).

## Figures and Tables

**Table 1:** Mediation analysis- direct and indirect effects.

	Coef	S.E	t	p
*Unrewarded Group*				
(A path) Encoding->Reactivation	1.125	0.145	7.738	<0.0001
(B path) Reactivation -> Day 3 memory strength	1.094	0.306	3.577	0.0060
(C path) Encoding -> Day 3 memory strength	1.041	0.207	5.02	0.0005
(C’ path)* Encoding -> Day 3 memory strength	−0.189	0.371	−0.507	0.6238
*Rewarded Group*				
(A path) Encoding->Reactivation	0.793	0.118	6.727	0.0001
(B path) Reactivation -> Day 3 memory strength	0.601	0.224	2.685	0.025
(C path) Encoding -> Day 3 memory strength	0.87	0.106	8.187	<0.0001
(C’ path)* Encoding -> Day 3 memory strength	0.393	0.196	2.004	0.076

^*^Effect of encoding on Day 3 memory strength, when controlling for reactivation.

**Table 2:** Mediation analysis- bootstrap results for indirect effects.

	Bootstrap results				Bias-corrected CI	
	Data	Boot	Bias	S.E	Lower	Upper
Unrewarded Group*	1.230	1.284	0.054	0.444	0.241	2.849
*Rewarded Group*	0.476	0.469	−0.007	0.183	−0.23	1.01

^*^Significant effect (CI is above 0).

## References

[b1] Agren T. . Disruption of reconsolidation erases a fear memory trace in the human amygdala. Science 337, 1550–2 (2012). 22997340 10.1126/science.1223006

[b2] Chan J. C. & LaPaglia J. A. Impairing existing declarative memory in humans by disrupting reconsolidation. Proc Natl Acad Sci USA 110, 9309–13 (2013). 23690586 10.1073/pnas.1218472110 PMC3677482

[b3] Nader K. & Hardt O. A single standard for memory: the case for reconsolidation. Nat Rev Neurosci 10, 224–234 (2009). 19229241 10.1038/nrn2590

[b4] Sandrini M., Censor N., Mishoe J. & Cohen L.G. Causal Role of Prefrontal Cortex in Strengthening of Episodic Memories through Reconsolidation. Current Biology 23, 2181–2184 (2013). 24206845 10.1016/j.cub.2013.08.045 PMC3824257

[b5] Sevenster D., Beckers T. & Kindt M. Prediction Error Governs Pharmacologically Induced Amnesia for Learned Fear. Science 339, 830–833 (2013). 23413355 10.1126/science.1231357

[b6] Haubrich J. . Reconsolidation allows fear memory to be updated to a less aversive level through the incorporation of appetitive information. Neuropsychopharmacology 40, 315–26 (2015). 25027331 10.1038/npp.2014.174 PMC4443944

[b7] Censor N., Horovitz S. G. & Cohen L. G. Interference with existing memories alters offline intrinsic functional brain connectivity. Neuron 81, 69–76 (2014). 24411732 10.1016/j.neuron.2013.10.042 PMC3894578

[b8] Lee J. L. C. Memory reconsolidation mediates the strengthening of memories by additional learning. Nat Neurosci 11, 1264–1266 (2008). 18849987 10.1038/nn.2205

[b9] Dudai Y. The neurobiology of consolidations, or, how stable is the engram? Annu Rev Psychol 55, 51–86 (2004). 14744210 10.1146/annurev.psych.55.090902.142050

[b10] Nader K., Schafe G. E. & Le Doux J. E. Fear memories require protein synthesis in the amygdala for reconsolidation after retrieval. Nature 406, 722–6 (2000). 10963596 10.1038/35021052

[b11] Abe M. . Reward Improves Long-Term Retention of a Motor Memory through Induction of Offline Memory Gains. Current Biology 21, 557–562 (2011). 21419628 10.1016/j.cub.2011.02.030 PMC3075334

[b12] Adcock R. A., Thangavel A., Whitfield-Gabrieli S., Knutson B. & Gabrieli J. D. Reward-motivated learning: mesolimbic activation precedes memory formation. Neuron 50, 507–517 (2006). 16675403 10.1016/j.neuron.2006.03.036

[b13] Shohamy D. & Adcock R. A. Dopamine and adaptive memory. Trends Cogn Sci 14, 464–72 (2010). 20829095 10.1016/j.tics.2010.08.002

[b14] Wittmann B. C. . Reward-related FMRI activation of dopaminergic midbrain is associated with enhanced hippocampus-dependent long-term memory formation. Neuron 45, 459–467 (2005). 15694331 10.1016/j.neuron.2005.01.010

[b15] Dayan E., Hamann J. M., Averbeck B. B. & Cohen L. G. Brain structural substrates of reward dependence during behavioral performance. J Neurosci 34, 16433–41 (2014). 25471581 10.1523/JNEUROSCI.3141-14.2014 PMC4252553

[b16] Hamann J. M., Dayan E., Hummel F. C. & Cohen L. G. Baseline frontostriatal-limbic connectivity predicts reward-based memory formation. Hum Brain Mapp 35, 5921–31 (2014). 25078102 10.1002/hbm.22594 PMC4883096

[b17] Wimmer G. E., Braun E. K., Daw N. D. & Shohamy D. Episodic Memory Encoding Interferes with Reward Learning and Decreases Striatal Prediction Errors. The Journal of Neuroscience 34, 14901–14912 (2014). 25378157 10.1523/JNEUROSCI.0204-14.2014 PMC4220024

[b18] Censor N., Dimyan M. A. & Cohen L. G. Modification of existing human motor memories is enabled by primary cortical processing during memory reactivation. Curr Biol 20, 1545–9 (2010). 20817532 10.1016/j.cub.2010.07.047 PMC2957647

[b19] Karni A. . Functional MRI evidence for adult motor cortex plasticity during motor skill learning. Nature 377, 155–158 (1995). 7675082 10.1038/377155a0

[b20] Walker M. P., Brakefield T., Hobson J. A. & Stickgold R. Dissociable stages of human memory consolidation and reconsolidation. Nature 425, 616–620 (2003). 14534587 10.1038/nature01930

[b21] MacKinnon D. P., Lockwood C. M. & Williams J. Confidence Limits for the Indirect Effect: Distribution of the Product and Resampling Methods. Multivariate behavioral research 39, 99–99 (2004). 20157642 10.1207/s15327906mbr3901_4 PMC2821115

[b22] Preacher K. J. & Hayes A. F. Asymptotic and resampling strategies for assessing and comparing indirect effects in multiple mediator models. Behav Res Methods 40, 879–91 (2008). 18697684 10.3758/brm.40.3.879

[b23] Cohen D. A. & Robertson E. M. Preventing interference between different memory tasks. Nature neuroscience 14, 953–955 (2011). 21706019 10.1038/nn.2840 PMC3144999

[b24] Hartley T. & Burgess N. Complementary memory systems: competition, cooperation and compensation. Trends Neurosci 28, 169–70 (2005). 15808348 10.1016/j.tins.2005.02.004

[b25] Poldrack R. A. . Interactive memory systems in the human brain. Nature 414, 546–50 (2001). 11734855 10.1038/35107080

[b26] Bouton M. E. Context, time, and memory retrieval in the interference paradigms of Pavlovian learning. Psychol Bull 114, 80–99 (1993). 8346330 10.1037/0033-2909.114.1.80

[b27] Bouton M. E., Winterbauer N. E. & Todd T. P. Relapse processes after the extinction of instrumental learning: renewal, resurgence, and reacquisition. Behav Processes 90, 130–41 (2012). 22450305 10.1016/j.beproc.2012.03.004 PMC3355659

[b28] Rescorla R. A. & Heth C. D. Reinstatement of fear to an extinguished conditioned stimulus. Journal of Experimental Psychology: Animal Behavior Processes 1, 88–96 (1975). 1151290

[b29] Bouton M. & Peck C. Spontaneous recovery in cross-motivational transfer (counterconditioning). Animal Learning & Behavior 20, 313–321 (1992).

[b30] Richardson R., Riccio D. C., Jamis M., Cabosky J. & Skoczen T. Modification of reactivated memory through “counterconditioning”. Am J Psychol 95, 67–84 (1982). 7125017

[b31] Tunstall B. J., Verendeev A. & Kearns D. N. A comparison of therapies for the treatment of drug cues in male rats: counterconditioning vs. extinction. Experimental and clinical psychopharmacology 20, 447–453 (2012). 23230857 10.1037/a0030593 PMC3716831

[b32] Exton-McGuinness M. T., Patton R. C., Sacco L. B. & Lee J. L. Reconsolidation of a well-learned instrumental memory. Learn Mem 21, 468–77 (2014). 25135195 10.1101/lm.035543.114 PMC4138356

[b33] Lee J. L. Reconsolidation: maintaining memory relevance. Trends Neurosci 32, 413–20 (2009). 19640595 10.1016/j.tins.2009.05.002 PMC3650827

[b34] Reichelt A. & Lee J. C. Over-expectation generated in a complex appetitive goal-tracking task is capable of inducing memory reconsolidation. Psychopharmacology 226, 649–658 (2013). 23239132 10.1007/s00213-012-2934-3

[b35] Kim G., Lewis-Peacock J. A., Norman K. A. & Turk-Browne N. B. Pruning of memories by context-based prediction error. Proceedings of the National Academy of Sciences 111, 8997–9002 (2014). 10.1073/pnas.1319438111 PMC4066528 24889631

[b36] Carbonell F. . Dopamine precursor depletion impairs structure and efficiency of resting state brain functional networks. Neuropharmacology 84, 90–100 (2014). 24412649 10.1016/j.neuropharm.2013.12.021

[b37] Wunderlich K., Smittenaar P. & Dolan R. J. Dopamine enhances model-based over model-free choice behavior. Neuron 75, 418–24 (2012). 22884326 10.1016/j.neuron.2012.03.042 PMC3417237

[b38] Strafella A. P., Paus T., Fraraccio M. & Dagher A. Striatal dopamine release induced by repetitive transcranial magnetic stimulation of the human motor cortex. Brain 126, 2609–15 (2003). 12937078 10.1093/brain/awg268

[b39] Deuker L. . Memory consolidation by replay of stimulus-specific neural activity. J Neurosci 33, 19373–83 (2013). 24305832 10.1523/JNEUROSCI.0414-13.2013 PMC6618788

[b40] Schlichting M. L. & Preston A. R. Memory reactivation during rest supports upcoming learning of related content. Proceedings of the National Academy of Sciences 111, 15845–15850 (2014). 10.1073/pnas.1404396111 PMC4226095 25331890

[b41] Sandrini M., Cohen L. G. & Censor N. Modulating reconsolidation: a link to causal systems-level dynamics of human memories. Trends in Cognitive Sciences 19, 475–482 (2015). 26170029 10.1016/j.tics.2015.06.002 PMC4523421

[b42] Korman M. . Daytime sleep condenses the time course of motor memory consolidation. Nature neuroscience 10, 1206–1213 (2007). 17694051 10.1038/nn1959

[b43] Anderson M. C., Bjork E. L. & Bjork R. A. Retrieval-induced forgetting: Evidence for a recall-specific mechanism. Psychonomic Bulletin & Review 7, 522–530 (2000). 11082860 10.3758/bf03214366

[b44] Anderson M. C., Bjork R. A. & Bjork E. L. Remembering can cause forgetting: retrieval dynamics in long-term memory. Journal of Experimental Psychology: Learning, Memory, and Cognition 20, 1063 (1994). 10.1037//0278-7393.20.5.1063 7931095

[b45] Celnik P., Paik N. J., Vandermeeren Y., Dimyan M. & Cohen L. G. Effects of combined peripheral nerve stimulation and brain polarization on performance of a motor sequence task after chronic stroke. Stroke 40, 1764–71 (2009). 19286579 10.1161/STROKEAHA.108.540500 PMC2692264

[b46] Censor N., Buch E. R., Nader K. & Cohen L. G. Altered Human Memory Modification in the Presence of Normal Consolidation. Cereb Cortex (2015). 10.1093/cercor/bhv180 PMC5004751 26271110

[b47] Wilcox R. The percentage bend correlation coefficient. Psychometrika 59, 601–616 (1994).

[b48] Pernet C. R., Wilcox R. R. & Rousselet G. A. Robust correlation analyses: false positive and power validation using a new open source Matlab toolbox. Frontiers in Psychology 3 (2013). 10.3389/fpsyg.2012.00606 PMC3541537 23335907

[b49] Preacher K. & Hayes A. SPSS and SAS procedures for estimating indirect effects in simple mediation models. Behavior Research Methods, Instruments, & Computers 36, 717–731 (2004). 10.3758/bf03206553 15641418

